# Updated Recommendations for Use of MenB-FHbp Serogroup B Meningococcal Vaccine — Advisory Committee on Immunization Practices, 2016

**DOI:** 10.15585/mmwr.mm6619a6

**Published:** 2017-05-19

**Authors:** Monica E. Patton, David Stephens, Kelly Moore, Jessica R. MacNeil

**Affiliations:** ^1^Division of Bacterial Diseases, National Center for Immunization and Respiratory Diseases, CDC; ^2^Advisory Committee on Immunization Practices Meningococcal Vaccines Work Group, Emory University School of Medicine, Atlanta, Georgia; ^3^Advisory Committee on Immunization Practices Meningococcal Vaccines Work Group, Tennessee Department of Health and Vanderbilt University School of Medicine, Nashville, Tennessee.

Two serogroup B meningococcal (MenB) vaccines are currently licensed for use in persons aged 10–25 years in the United States. The two vaccines are MenB-FHbp (Trumenba, Pfizer, Inc.) (*1*) and MenB-4C (Bexsero, GlaxoSmithKline Biologicals, Inc.) (*2*). In February 2015, the Advisory Committee on Immunization Practices (ACIP) recommended use of MenB vaccines among certain groups of persons aged ≥10 years who are at increased risk for serogroup B meningococcal disease* (Category A) (*3*), and in June 2015, ACIP recommended that adolescents and young adults aged 16–23 years may be vaccinated with MenB vaccines to provide short-term protection against most strains of serogroup B meningococcal disease (Category B^†^) (*4*). Consistent with the original Food and Drug Administration (FDA) licensure for the two available MenB vaccines, ACIP recommended either a 3-dose series of MenB-FHbp or a 2-dose series of MenB-4C. Either MenB vaccine can be used when indicated; ACIP does not state a product preference. The two MenB vaccines are not interchangeable; the same vaccine product must be used for all doses in a series. In April 2016, changes to the dosage and administration of MenB-FHbp were approved by FDA to allow for both a 2-dose series (administered at 0 and 6 months) and a 3-dose series (administered at 0, 1–2, and 6 months) (*5*,*6*). In addition, the package insert now states that the choice of dosing schedule depends on the patient’s risk for exposure and susceptibility to serogroup B meningococcal disease. These recommendations are regarding use of the 2- and 3-dose schedules of MenB-FHbp vaccine (Trumenba) and replace previous ACIP recommendations for use of MenB-FHbp vaccine published in 2015 (*3*,*4*). Recommendations regarding use of MenB-4C (Bexsero) are unchanged (*3*,*4*).

## Methods

The ACIP Meningococcal Vaccines Work Group identified studies of the comparative immunogenicity, safety, and antibody persistence of 2- and 3-dose schedules of MenB-FHbp vaccine by consulting with the manufacturer and searching PubMed using the search terms “meningococcal serogroup B vaccine,” “Trumenba,” and “MenB-FHbp.” One relevant published clinical trial (*7*) and unpublished data from the same trial (Pfizer, unpublished data^§^) were identified that compared immunogenicity and safety of 2- and 3-dose schedules of MenB-FHbp vaccine. Additionally, unpublished data were identified (Pfizer, unpublished data^¶^) for participants in the same trial who were enrolled in an extension study designed to evaluate antibody persistence annually for 48 months and response to a single booster dose approximately 48 months after the primary series. The Work Group reviewed published and unpublished immunogenicity and safety data from the clinical trial and unpublished antibody persistence data and booster dose response data. The type and quality of evidence supporting the use of MenB vaccines in adolescents and young adults (including college students) and persons at increased risk for serogroup B meningococcal disease were evaluated previously using the Grading of Recommendations, Assessment, Development, and Evaluation (GRADE) framework (*3*,*4*,*8*,*9*). Summaries of the Work Group discussions and data reviewed were presented to ACIP in June and October 2016, and recommendations were approved by the voting ACIP members at the October 2016 meeting (detailed meeting minutes are available at https://www.cdc.gov/vaccines/acip/meetings/meetings-info.html).

## MenB-FHbp Immunogenicity

Previous ACIP policy statements have described the assessments of MenB-FHbp immunogenicity data for persons aged ≥10 years that supported FDA licensure (*3*,*4*,*8*,*9*). The immunogenicity of 3-dose versus 2-dose MenB-FHbp schedules in adolescents and young adults was evaluated in a clinical trial conducted in Europe among 1,450 persons aged 11–18 years (*7*) (Pfizer, unpublished data). Participants were randomly assigned to one of five groups. Group 1 received MenB-FHbp at months 0, 1, and 6 and received a saline injection at month 2; group 2 received MenB-FHbp at months 0, 2, and 6 and saline at month 1; group 3 received MenB-FHbp at months 0 and 6 and saline at months 1 and 2; group 4 received MenB-FHbp at months 0 and 2 and saline at months 1 and 6; group 5 received MenB-FHbp at months 2 and 6 and saline at months 0 and 1 (referred to as 0, 4 months below). Serum bactericidal antibody activity, measured using human complement (hSBA) was used as a correlate of protection to assess vaccine immunogenicity (*10*,*11*). Immunogenicity in the trial was assessed as the percentage of subjects who achieved an hSBA titer greater than or equal to the lower limit of quantification of the assay (hSBA titer ≥1:8) to each of the four selected serogroup B meningococcal strains tested (*7*,*12*). For purposes of this evaluation, immunogenicity was assessed as the proportion of subjects who achieved an hSBA titer ≥1:8** to all four selected strains tested (composite response) (Pfizer, unpublished data).

Among the 3-dose schedules evaluated, 83.1% of subjects in group 1 (0, 1, 6 months) and 81.7% of subjects in group 2 (0, 2, 6 months) had a composite response (hSBA titer ≥1:8) to all four strains tested at 1 month following the third dose (Table) (Pfizer, unpublished data). Among the 2-dose schedules, group 3 (0, 6 months) had the highest percent of responders, 73.5%; 58.9% of subjects in group 5 (0, 4 months) and 56.8% of subjects in group 4 (0, 2 months) had a composite response to all four strains tested at 1 month following the second dose. In addition, whereas geometric mean antibody titers (GMTs) were higher to all four strains tested among subjects who received 3 doses compared with those who received 2 doses, group 3 (0, 6 months) had the highest GMTs among all 2-dose schedules (*7*).

**TABLE:** Percentage of persons* aged 11–18 years who achieved an hSBA titer ≥1:8^†^ against all four selected serogroup B meningococcal strains tested^§^ (composite response) at 1 month (m) following completion of a 3-dose (0, 1, 6 months; and 0, 2, 6 months) or 2-dose (0, 6 months; 0, 2 months; and 0, 4 months) series of MenB-FHbp

Series	Group	hSBA titer ≥1:8^†^ against all four serogroup B strains^§^ (%)	95% confidence interval
3-dose series	Group 1 (0, 1, 6 m)	83.1	78.6–86.9
	Group 2 (0, 2, 6 m)	81.7	77.3–85.7
2-dose series	Group 3 (0, 6 m)	73.5	68.5–78.1
	Group 4 (0, 2 m)	56.8	52.5–61.0
	Group 5 (0, 4 m)	58.9	49.0–68.3

**Abbreviation:** hSBA = serum bactericidal antibody activity, measured using human complement.

* Number of subjects in the evaluable immunogenicity population = 1,450.

^†^ hSBA titer ≥1:16 for the serogroup B strain expressing FHbp (factor H binding protein) A22.

^§^ Serogroup B meningococcal strains expressing FHbp of subfamily A (A22, A56) and subfamily B (B24, B44).

## MenB-FHbp Antibody Persistence

**FIGURE 1:**
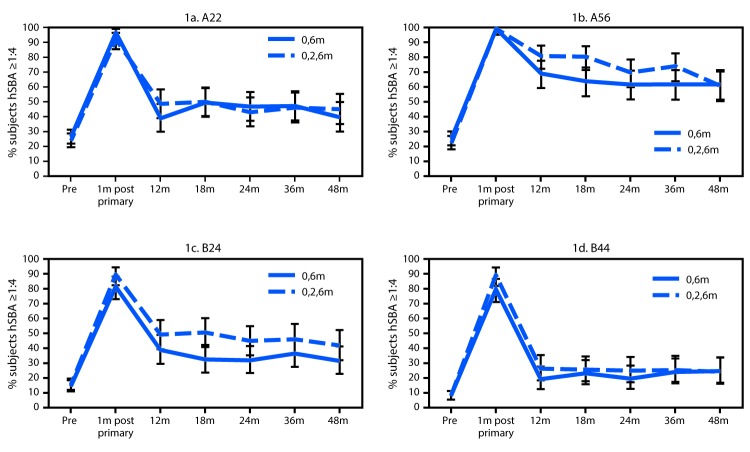
Persistence of hSBA responses ≥1:4* against four selected serogroup B meningococcal strains**^†^** in subjects aged 11–18 years,**^§^** up to 48 months (m) after completion of a 2-dose (0, 6 months) or 3-dose (0, 2, 6 months) series of MenB-FHbp **Abbreviation:** hSBA = serum bactericidal antibody activity, measured using human complement. * Expressed as a percentage, with error bars representing 95% confidence intervals. ^†^ Serogroup B meningococcal strains expressing FHbp (factor H binding protein) of subfamily A (A22, A56) or subfamily B (B24, B44). ^§^ Number of subjects for persistence time points: 0, 6 m = 99–116; 0, 2, 6 m = 92–114.

**FIGURE 2:**
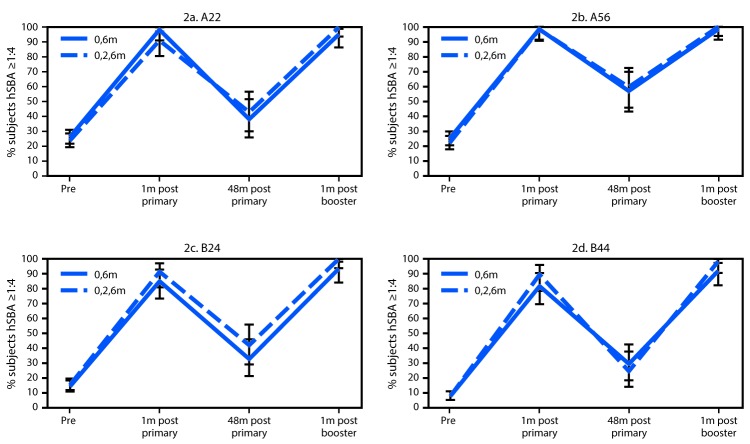
Persistence of hSBA responses ≥1:4* and GMTs^†^ against four selected serogroup B meningococcal strains^§^ at 48 months (m) in subjects aged 11–18 years^¶^ after completion of a 2-dose (0, 6 months) or 3-dose (0, 2, 6 months) series of MenB-FHbp, and hSBA responses ≥1:4 and GMTs to a booster dose of MenB-FHbp at approximately 48 months after primary vaccination **Abbreviations:** GMTs = geometric mean antibody titers; hSBA = serum bactericidal antibody activity, measured using human complement. * Expressed as a percentage, with error bars representing 95% confidence intervals. ^†^ GMTs were as follows. **A22** **(0, 6 m)**: Pre 6.4, 1m post primary 55.8, 48m post primary 15.3, 1m post booster 140.0; **(0, 2, 6m)**: Pre 6.3, 1m post primary 59.5, 48m post primary 15.4, 1m post booster 119.1. **A56** **(0, 6m)**: Pre 6.7, 1m post primary 143.1, 48m post primary 15.8, 1m post booster 358.0; **(0, 2, 6m)**: Pre 6.1, 1m post primary 191.2, 48m post primary 17.4, 1m post booster 370.8. **B24** **(0, 6m)**: Pre 5.0, 1m post primary 29.2, 48m post primary 7.8, 1m post booster 86.0; **(0, 2, 6m)**: Pre 5.1, 1m post primary 30.5, 48m post primary 9.1, 1m post booster 80.3. **B44 (0, 6m)**: Pre 4.5, 1m post primary 35.5, 48m post primary 5.3, 1m post booster 84.6; **(0, 2, 6m)**: Pre 4.5, 1m post primary 50.2, 48m post primary 5.3, 1m post booster 117.6. ^§^ Serogroup B meningococcal strains expressing FHbp (factor H binding protein) of subfamily A (A22, A56) or subfamily B (B24, B44). ^¶^ Number of subjects for persistence time points: 0, 6 m = 58–62; 0, 2, 6 m = 57–58.

Antibody persistence data through 48 months and response to a single booster dose at approximately 48 months were evaluated for participants aged 11–18 years in the clinical trial described who also enrolled in an extension study (Pfizer, unpublished data). The percentage of subjects with protective titers to all four of the serogroup B meningococcal strains tested was evaluated at 1, 12, 18, 24, 36, and 48 months following completion of the aforementioned 2-dose and 3-dose schedules and at 1 month following the booster dose at 48 months. An hSBA titer ≥1:4 was considered protective, a lower level of activity than the hSBA titer of ≥1:8 used to assess immunogenicity. Among subjects enrolled in the extension study who received the 2-dose (0, 6 month) schedule and the 3-dose (0, 2, 6 month) schedule, the percentages of subjects with protective hSBA titers to the four selected strains did not statistically differ at any time point (Figure 1). At 1 month following completion of the primary series, 78.9%–98.9% of subjects who received the 2-dose schedule and 86.5%–99.1% of subjects who received the 3-dose schedule had protective hSBA titers to the four selected strains. For both groups, the percentage of subjects with protective antibodies declined sharply at 12 months after completion of the primary series and remained stable through 48 months after vaccination (Figure 1). The hSBA responses and GMTs following a single booster dose at approximately 48 months after primary vaccination for the group that received the 2-dose schedule were not statistically different from those of the group that received the 3-dose schedule (Figure 2).

## MenB-FHbp Safety

MenB-FHbp safety data have been reported previously (*3*,*4*,*8*,*9*). No significant increased risk for serious adverse events has been identified among >4,250 subjects aged 10–25 years in seven clinical trials who received at least 1 dose of MenB-FHbp (*4*,*6*,*8*,*9*). The most common adverse reactions observed in the 7 days after receipt of MenB-FHbp were pain at the injection site (≥85% of subjects), fatigue (≥40%), headache (≥35%), myalgia (≥30%), and chills (≥15%). Safety and tolerability profiles were similar among subjects aged 11–18 years who were randomly assigned either a 3-dose or 2-dose series of MenB-FHbp (*6*,*7*).

## ACIP Recommendations

These recommendations are regarding use of the 2- and 3-dose schedules of MenB-FHbp vaccine (Trumenba) and replace previous ACIP recommendations for use of MenB-FHbp vaccine published in 2015 (*3*,*4*). Recommendations regarding use of MenB-4C (Bexsero) are unchanged (*3*,*4*).

**Persons aged ≥10 years at increased risk for serogroup B meningococcal disease (Category A recommendation).** For persons at increased risk for meningococcal disease and for use during serogroup B meningococcal disease outbreaks, 3 doses of MenB-FHbp should be administered at 0, 1–2, and 6 months to provide earlier protection and maximize short-term immunogenicity. However, if the second dose of MenB-FHbp is administered at an interval of ≥6 months, a third dose does not need to be administered.

**Adolescents and young adults aged 16–23 years (Category B recommendation).** When given to healthy adolescents who are not at increased risk for meningococcal disease, 2 doses of MenB-FHbp should be administered at 0 and 6 months. If the second dose of MenB-FHbp is administered earlier than 6 months after the first dose, a third dose should be administered at least 4 months after the second dose.

## CDC Guidance for Use

There are two MenB vaccines licensed for use in the United States among persons aged 10–25 years. Either MenB vaccine can be used when indicated; ACIP does not state a product preference. The two MenB vaccines are not interchangeable; the same vaccine product must be used for all doses in a series. The minimum interval between any 2 doses of MenB vaccine is 4 weeks. On the basis of available data and expert opinion, MenB-FHbp or MenB-4C may be administered concomitantly with other vaccines indicated for this age, but at a different anatomic site, if feasible. ACIP will consider MenB vaccine booster doses for persons at increased risk for serogroup B meningococcal disease as data become available.

No randomized controlled clinical trials have been conducted to evaluate the use of MenB vaccines in pregnant or lactating women. As stated in previous ACIP reports on MenB vaccines, vaccination should be deferred in women known to be pregnant or lactating unless the woman is at increased risk for serogroup B meningococcal disease, and, after consultation with her health care provider, the benefits of vaccination are considered to outweigh the potential risks. Additional information for health care providers and parents can be found at https://www.cdc.gov/meningococcal.

## Precautions and Contraindications

Before administering serogroup B meningococcal vaccines, health care providers should consult the package inserts for precautions, warnings, and contraindications (*6*,*13*). Adverse events occurring after administration of any vaccine should be reported to the Vaccine Adverse Event Reporting System (VAERS). Reports can be submitted to VAERS online, by fax, or by mail. Additional information about VAERS is available by telephone (1–800–822–7967) or online (https://vaers.hhs.gov).

**Summary**

**What is currently recommended?**

Two serogroup B meningococcal (MenB) vaccines are currently licensed for use among persons aged 10–25 years in the United States: MenB-FHbp (Trumenba) and MenB-4C (Bexsero). The Advisory Committee on Immunization Practices (ACIP) currently recommends routine use of MenB vaccines among persons aged ≥10 years who are at increased risk for serogroup B meningococcal disease (Category A recommendation), including persons who have persistent complement component deficiencies; persons who have anatomic or functional asplenia; microbiologists who routinely are exposed to isolates of *Neisseria meningitidis*; and persons identified to be at increased risk because of a serogroup B meningococcal disease outbreak. Adolescents and young adults aged 16–23 years may also be vaccinated with MenB vaccines to provide short-term protection against most strains of serogroup B meningococcal disease (Category B recommendation). Consistent with the original Food and Drug Administration (FDA) licensure for the MenB vaccines, ACIP recommended either a 3-dose series of MenB-FHbp or a 2-dose series of MenB-4C. Either MenB vaccine can be used when indicated; however, they are not interchangeable, and the same product must be used for all doses.

**Why are the recommendations being modified now?**

Changes to the dosage and administration of MenB-FHbp were approved by FDA to include both a 3-dose series (administered at 0, 1–2, and 6 months) and a 2-dose series (administered at 0 and 6 months).

**What are the new recommendations?**

These updated recommendations are regarding use of the 2- and 3-dose schedules of MenB-FHbp vaccine (Trumenba). For persons at increased risk for meningococcal disease and for use during serogroup B meningococcal disease outbreaks, ACIP recommends that 3 doses of MenB-FHbp be administered at 0, 1–2, and 6 months. When given to healthy adolescents who are not at increased risk for meningococcal disease, ACIP recommends that 2 doses of MenB-FHbp should be administered at 0 and 6 months. Recommendations regarding use of MenB-4C vaccine (Bexsero) are unchanged. Either MenB vaccine can be used when indicated; however, they are not interchangeable, and the same product must be used for all doses in a series.

## Footnotes

* Persons with persistent complement component deficiencies (including inherited or chronic deficiencies in C3, C5–C9, properdin, factor D, factor H, or who are taking eculizumab [Solaris]); persons with anatomic or functional asplenia (including sickle cell disease); microbiologists routinely exposed to isolates of *Neisseria meningitidis*; persons identified as at increased risk because of a serogroup B meningococcal disease outbreak.

† Category A recommendations are made for all persons in an age- or risk-factor-based group. Category B recommendations are made for individual clinical decision making. https://www.cdc.gov/vaccines/acip/recs/grade/about-grade.html.

§ Unpublished data, study B1971012, https://clinicaltrials.gov/ct2/show/NCT01299480, ACIP meeting October 2016.

¶ Unpublished data, study B1971033, https://clinicaltrials.gov/ct2/show/NCT01543087, ACIP meeting October 2016.

** Lower limit of quantification for the MenB strain expressing FHbp A22 was hSBA titer ≥1:16.
